# The effect of two β-alanine dosing strategies on 30-minute rowing performance: a randomized, controlled trial

**DOI:** 10.1186/s12970-018-0266-3

**Published:** 2018-12-18

**Authors:** Liam Beasley, Lee Smith, Jose Antonio, Dan Gordon, James Johnstone, Justin Roberts

**Affiliations:** 1 Cambridge Centre for Sport and Exercise Sciences, Anglia Ruskin University, Cambridge, UK; 2 College of Health Care Sciences, Nova Southeastern University, Florida, USA

**Keywords:** Beta-alanine, Rowing, Endurance, Exercise performance, Nutrition

## Abstract

**Background:**

β-alanine (βA) supplementation has been shown to increase intramuscular carnosine content and subsequent high-intensity performance in events lasting < 4 minutes (min), which may be dependent on total, as opposed to daily, dose. The ergogenic effect of βA has also been demonstrated for 2000-m rowing performance prompting interest in whether βA may be beneficial for sustained aerobic exercise. This study therefore investigated the effect of two βA dosing strategies on 30-min rowing and subsequent sprint performance.

**Methods:**

Following University Ethics approval, twenty-seven healthy, male rowers (age: 24 ± 2 years; body-height: 1.81 ± 0.02 m; body-mass: 82.3 ± 2.5 kg; body-fat: 14.2 ± 1.0%) were randomised in a double-blind manner to 4 weeks of: i) βA (2.4 g·d^− 1^, βA1); ii) matched total βA (4.8 g on alternate days, βA2); or iii) cornflour placebo (2.4 g·d^− 1^, PL). Participants completed a laboratory 30-min rowing time-trial, followed by 3x30-seconds (s) maximal sprint efforts at days 0, 14 and 28 (T1-T3). Total distance (m), average power (W), relative average power (W·kg^− 1^), cardio-respiratory measures and perceived exertion were assessed for each 10-min split. Blood lactate ([La-]_b_ mmol·L^− 1^) was monitored pre-post time-trial and following maximal sprint efforts. A 3-way repeated measures ANOVA was employed for main analyses, with Bonferonni post-hoc assessment (*P* ≤ 0.05).

**Results:**

Total 30-min time-trial distance significantly increased from T1-T3 within βA1 only (7397 ± 195 m to 7580 ± 171 m, *P* = 0.002, ƞp^2^ = 0.196), including absolute average power (194.8 ± 18.3 W to 204.2 ± 15.5 W, *P* = 0.04, ƞp^2^ = 0.115) and relative average power output (2.28 ± 0.15 W·kg^− 1^ to 2.41 ± 0.12 W·kg^− 1^, *P* = 0.031, ƞp^2^ = 0.122). These findings were potentially explained by within-group significance for the same variables for the first 10 min split (*P* ≤ 0.01), and for distance covered (*P* = 0.01) in the second 10-min split. However, no condition x time interactions were observed. No significant effects were found for sprint variables (*P* > 0.05) with comparable values at T3 for mean distance (βA1: 163.9 ± 3.8 m; βA2: 161.2 ± 3.5 m; PL: 162.7 ± 3.6 m), average power (βA1: 352.7 ± 14.5 W; βA2: 342.2 ± 13.5 W; PL: 348.2 ± 13.9 W) and lactate (βA1: 10.0 ± 0.9 mmol·L^− 1^; βA2: 9.2 ± 1.1 mmol·L^− 1^; PL: 8.7 ± 0.9 mmol·L^− 1^).

**Conclusions:**

Whilst daily βA may confer individual benefits, these results demonstrate limited impact of βA (irrespective of dosing strategy) on 30-min rowing or subsequent sprint performance. Further investigation of βA dosage > 2.4 g·d^− 1^ and/or chronic intervention periods (> 4–8 weeks) may be warranted based on within-group observations.

## Introduction

β-alanine (βA) first gained popularity within the athletic population during the mid-2000’s following the inaugural work of Harris and colleagues [1]. Numerous research has since demonstrated that βA supplementation (1.6–6.4 g·day^− 1^ for ≥28 days) augments the naturally occurring histidine dipeptide, carnosine (*β-alanyl-L-histidine*) within human muscle tissue [2–5]. As carnosine acts as a physico-chemical buffering agent (with an imidazole ring pK_a_ of 6.83, and relative similarity to intracellular pH (6.5) [6–8]), βA supplementation was recently reported as producing small, yet significant performance improvements (effect size: 0.210; 95% CI: 0.057, 0.362) across a range of short (60–240 s) duration events [9] including cycling sprint performance [10], judo bouts [11], 800 m sprinting [12] and 1000 m rowing splits [13] in club-level through to well training men.

Beyond these short duration (60–240 s) bouts the magnitude of improvement appears to diminish. However, a recent meta-analysis based on 40 individual studies involving 1461 participants indicated that the predominant use of incremental tests in many studies may potentially bias this finding based on assessment of exercise capacity as opposed to aerobic performance [9]. Additionally, in a recent International Society of Sports Nutrition (ISSN) Position Stand, Trexler et al [14] noted an apparent dearth of βA research investigating performance during endurance events (> 25-min), with inference that βA may ergogenically support training adaptations to sustained efforts typically employed in aerobically dominated events [15–18].

Specific to rowing, previous research has highlighted a significant correlation between muscle carnosine content and performances over multiple distances (100, 500, 2000 and 6000 m) [19]. It is therefore feasible that short-term βA supplementation could support prolonged training bouts or enhance > 2000 m performance, particularly in non-elite rowers or those with lower initial muscle carnosine levels [20–22]. With the high levels of acidity brought about as a bi-product of anaerobic glycolysis, and subsequent elevated blood lactate production observed in competitive rowers, the buffering potential of carnosine could facilitate higher power output throughout an endurance bout/race, or as part of a final sprint [19, 23]. However, such hypotheses rely on assumptions that perhaps over-simplify the mechanistic effects of βA, including improved calcium sensitivity [24], enhanced antioxidant capacity and reduced oxidative damage [25, 26].

In terms of dosing strategies, βA intakes from 1.6 g·day^− 1^ to 12 g·day^− 1^ [2, 27] for ≥2-weeks have been shown to significantly increase muscle carnosine content (with typically recommended levels of 2–6 g·day^− 1^ [1, 4, 7, 10, 28]). However, research has also highlighted that muscle carnosine content correlates significantly with total dose (grams) of βA consumed [2, 29] irrespective of baseline carnosine levels or daily intake. This raises an important question as to whether βA needs to be consumed daily, or whether ergogenic benefits are based on total dose provided in a given timeframe [2]. This is particularly relevant considering a recent survey in which 61% of Australian team sport athletes used βA as part of their training, yet only 35% understood the mechanistic benefits, and 50% consumed less than half of the commercially recommended dose [30].

The aim of the current study was therefore to assess the efficacy of two βA strategies (daily versus alternate day intake for 4 weeks) on 30-min rowing time-trial performance and subsequent anaerobic sprint bouts in healthy, male rowers. It was hypothesised that βA would significantly enhance endurance performance irrespective of dosing strategy.

## Materials/ methods

### Study design and participants

A randomised, double-blind, placebo controlled, parallel design was employed for this study. The study was conducted in accordance with the Declaration of Helsinki, and the protocol was approved by the Faculty of Science and Technology Ethics Committee, Anglia Ruskin University (Project Number: FST/FREP/15/591). A priori sample size using G*power software (α = 0.05 and 1-β = 0.90) based on performance data from Ducker et al. [13] estimated a total sample population of 27 participants. Participants were required to be healthy, male rowers with > 6 months training experience (including familiarity with 30- min time-trial sessions), and actively training > 3 times per week at the point of inclusion.

Informed consent was obtained from all individual participants prior to study inclusion. All participants satisfactorily completed a health screen questionnaire, and had no known history of blood related disorders, cardiovascular or metabolic abnormalities; or recent viral infections or injuries, which would prevent them from maintaining habitual training sessions or taking part in laboratory time-trials. Individuals at risk of lowered baseline carnosine, as a result of dietary restriction of animal products (vegan/vegetarians), were excluded from participation [31]. All participants were required not to be taking any medication / supplementation in the previous 3 months which could interfere with the study parameters, and in particular, complete abstinence (> 6 months) from βA-containing supplements specifically due to the slow washout rate previously reported for muscle carnosine content [32, 33]. Thirty male individuals volunteered for study inclusion. However, 3 were excluded from final analyses due to non-compliance with protocol requirements. Participant characteristics are displayed in Table 1.

**Table 1:** Participant characteristics and baseline measurements

Variable	All Participants (*n* = 27)	βA1 (*n* = 9)	βA2 *(n* = 9)	PL (*n* = 9)
Age (years)	24 ± 5	20 ± 6	24 ± 5	23 ± 1
Body- height (m)	1.81 ± 0.01	1.81 ± 0.02	1.81 ± 0.02	1.82 ± 0.02
Body-mass (kg)	82.4 ± 1.4	84.4 ± 3.1	80.3 ± 1.8	82.4 ± 2.6
Body-fat (%)	14.2 ± 0.6	15.1 ± 1.0	12.7 ± 1.1	14.8 ± 0.6
Fat-free mass (kg)	70.6 ± 1.2	71.5 ± 2.3	70.0 ± 1.6	70.3 ± 2.4
Experience (yrs)	2.0 ± 0.3	1.7 ± 0.5	2.0 ± 0.4	2.3 ± 0.6

Table 1 outlines baseline participant characteristics. Data are presented as M ± SE. *βA1* daily beta-alanine strategy, *βA2* alternate day strategy, *PL* placebo. No significant between-group differences reported.

### Procedures

All testing took place within the Cambridge Centre for Sport and Exercise Sciences, Human Physiology Laboratory, Anglia Ruskin University, Cambridge under thermal neutral conditions (temperature:17.1–19.3 °C; humidity: 20–38%; barometric pressure: 993–1028 mbar). Following pre-familiarization with the laboratory equipment and test protocol, participants were required to attend the laboratory on 3 occasions across the intervention period (days 0, 14 and 28; T1-T3) at the same time of day for each participant to minimise diurnal variance. Participants were instructed to avoid strenuous exercise, and refrain from consuming caffeinated or alcohol containing products at least 24 hours prior to each laboratory visit. Participants were requested to arrive acutely fasted (i.e. no food within 3 hours of assessment and maintain habitual hydration patterns) with last consumption of fluid (~ 0.5 L water) 1 hour prior to assessment to standardise procedures.

Upon arrival, body mass (Seca 780, Hamburg, Germany), height (Seca 200 stadiometer, Hamburg, Germany) and estimated body composition (4-site skinfold measures in triplicate) were assessed by the same researcher. Following a 5-min seated period, 2-min baseline expired air samples were collected via the Douglas bag method [34] and analysed for percentage O_2_ and CO_2_, using a Servoflex MINIMP 5200 gas analyser (Servomex Group Ltd., Crowborough, UK). Total Douglas bag volume was measured using a dry gas meter (Harvard Apparatus, Holliston, USA), with sample temperature recorded during volume measurement. Heart rate (HR) was recorded via radio-telemetry (T-31, Polar Electro Ltd., Kempele, Finland), and 20 μl capillarised fingertip blood samples were collected for analysis of blood lactate [La^−^]_b_ (Biosen C_Line, EKF Diagnostics, Cardiff, UK).

### Rowing ergometer time trials (TT)

All time-trials were conducted on the same Concept 2 air braked rowing ergometer (Concept 2, Nottingham, UK) with resistance set at 5 for standardisation. Participants undertook a self-paced, continuous warm-up at 100 W for 5-min, after which the unit display was modified to display time remaining to minimise extraneous influences on pacing strategy [35]. Participants were instructed to row for maximal distance in 30- min at self-selected pace. To minimise effects of data collection on performance, expired air, rated perceived exertion (RPE [36]) and HR were assessed during the final minute of each 10-min split; along with split distance covered (m) and mean power output (W).Verbal encouragement was provided by the same tester at the end of each collection period in a standard manner. End-point [La^−^]_b_ was assessed following the final collection period only.

### Sprint efforts post-TT

Following a standardised 5-min inactive rest period, participants completed three 30-s maximal sprint efforts with 60-s inactive recovery in between. [La^−^]_b_, RPE and performance data (distance rowed (m), average power (W)) were recorded on completion of each sprint. At the mid-point of each sprint, standardised verbal encouragement was given to all participants to promote maximal engagement.

### Supplemental treatments

Participants were randomly assigned (using a random number generator – https://www.randomizer.org/) in a double-blind manner to 4 weeks of: i) crystalline βA (2.4 g·d^− 1^ Bulk Powders®, UK; βA1); ii) matched total βA (4.8 g on alternate days, βA2); or iii) cornflour placebo (2.4 g·d^− 1^, PL). These daily doses were selected to minimise the risk of paraesthesia and thus the potential to affect the double blinding process. Furthermore, the matched average daily dose of 2.4 g∙day^− 1^ was deemed appropriate given previous research demonstrating increased muscle carnosine concentrations at lower daily doses (1.6 g∙day^− 1^) [2]. All products were manually weighed under laboratory conditions for accuracy and capsulated in size 00 capsules (hydroxypropyl methylcellulose). Once weighed all capsules were placed in food safe containers before an independent researcher recorded and randomized all batches to ensure double blinding procedures. All participants received capsules on a 14-day basis, along with a daily adherence diary to monitor compliance. To limit both the occurrence and severity of potential paraesthesia symptoms, standard instructions were provided to participants to consume one (800 mg) capsule daily with breakfast before repeatedly consuming (800 mg) capsules every 3 hours until required dosage was met, in accordance with previous research [37, 38]. Upon completion of each 14-day period participants were instructed to return any remaining capsules as a secondary measure of compliance.

### Dietary intake

Prior to baseline measures, and throughout the intervention, participants were requested to maintain habitual dietary intake and exercise patterns, and record using standard food/activity diaries (following individual guidance in diary collation, with emphasis on meal content, portion size and weight and fluid intake). In particular, participants were requested to refrain from introducing atypical foods during the intervention period. Diaries were comprehensively checked by the research team at each visit, with dietary analyses undertaken using Nutritics software (version 3.74 professional edition, Nutritics Ltd., Co. Dublin, Ireland). No differences were reported between groups for macronutrients and/or energy intake (Table 2), demonstrating general dietary compliance prior to testing sessions.

**Table 2:** Mean group dietary intake across the intervention period (T1-T3)

Variable	βA1	βA2	PL
Energy (kcal^.^kg^-1.^d^–1^)			
T1	27.73 ± 1.49	25.32 ± 1.23	29.00 ± 1.88
T2	28.34 ± 1.25	23.00 ± 1.16	26.46 ± 1.99
T3	25.40 ± 2.09	25.78 ± 1.19	28.35 ± 1.63
Protein (g kg^-1.^d^–1^)			
T1	1.91 ± 0.33	1.69 ± 10.15	1.84 ± 0.26
T2	1.71 ± 0.23	1.63 ± 0.11	1.82 ± 0.22
T3	1.85 ± 0.34	1.85 ± 0.18	1.82 ± 0.32
Carbohydrate (g^.^kg^-1.^d^–1^)			
T1	2.64 ± 0.12	2.20 ± 0.16	2.69 ± 0.32
T2	2.95 ± 0.26	2.22 ± 0.22	2.44 ± 0.33
T3	2.23 ± 0.14	2.26 ± 0.18	2.64 ± 0.34
Fat (g^.^kg^-1.^d^–1^)			
T1	1.02 ± 0.11	1.06 ± 0.13	1.17 ± 0.12
T2	0.97 ± 0.14	0.82 ± 0.09	1.06 ± 0.18
T3	0.97 ± 0.12	1.02 ± 0.11	1.13 ± 0.17

Table 2 shows relative dietary intake for both group and time. No significant differences reported within or between groups.

### Statistical analyses

Statistical analyses were performed using SPSS (v24, Chicago, USA). Normal distribution of data was assessed via a Shapiro-Wilks test [39]. A 3-way repeated measures ANOVA was employed for main analyses (including effect size (partial eta squared; ηp^2^)), with Bonferonni post-hoc assessment where applicable. Where pertinent, a one-way ANOVA with Bonferroni post hoc assessment was utilised to evaluate within treatment effects (e.g. baseline variables and resting measures). An alpha level of ≤0.05 was employed for statistical significance. Data are reported as means ± SE.

## Results

### Baseline characteristics and resting measures

Intervention groups were matched for age (yrs), rowing experience (yrs), body-height (m), body-mass (kg) and body-fat (%) at baseline (Table 1). Non significant differences (*P >* 0.05*)* were reported between groups for baseline resting HR (b·min^− 1^) (βA1: 66 ± 3, βA2: 62 ± 3, PL: 60 ± 2), [La^−^]_b_ (mmol·L^− 1^) (βA1: 1.4 ± 0.1, βA2: 1.4 ± 0.1, PL: 1.3 ± 0.1), absolute VO_2_ (L·min^− 1^) (βA1: 0.34 ± 0.04, βA2: 0.34 ± 0.02, PL: 0.37 ± 0.03) or relative VO_2_ (ml·kg^− 1^·min^− 1^) (βA1: 4.08 ± 0.40, βA2: 4.11 ± 0.24, PL: 4.28 ± 0.28).

### Time-trial performance measures

#### Overall distance

**Fig. 1:**
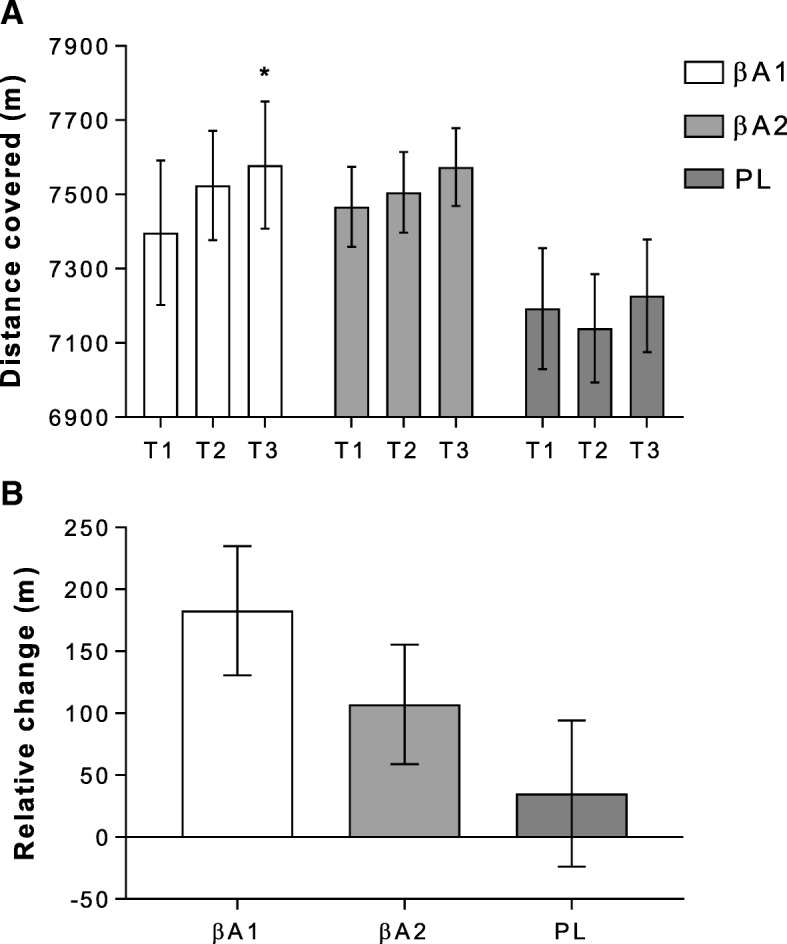
demonstrates effect of beta-alanine (βA) interventions on a) absolute and b) relative 30-min rowing time trial distance. Abbreviations: βA1 – daily intervention; βA2 – alternate day; PL – placebo. * represents significant difference, from T1, within group only

Data for distance rowed (m) during the 30-min time trial are shown as absolute a) and relative b) values in Fig. 1. No group x time interactions were shown for overall 30-min time trial performance (F = 1.50, *P* = 0.22, ηp^2^ = 0.11). A significant effect was shown for time only (F = 5.87, *P* = 0.005 ηp^2^ = 0.20), with βA1 distance increasing from 7397 ± 195 m at T1 to 7580 ± 171 m by T3 only (*P* = 0.002, Fig. 1). This represented a 2.45% absolute improvement in performance within-group only. When expressed relatively (T1-T3), despite a 2.60% increase in distance covered (183 ± 52 m) with βA1, and a 1.50% increase in distance covered (107 ± 48 m) with βA2, no significant between group differences were noted (F = 1.91, *P* = 0.17).

#### Power: Weight

**Fig. 2:**
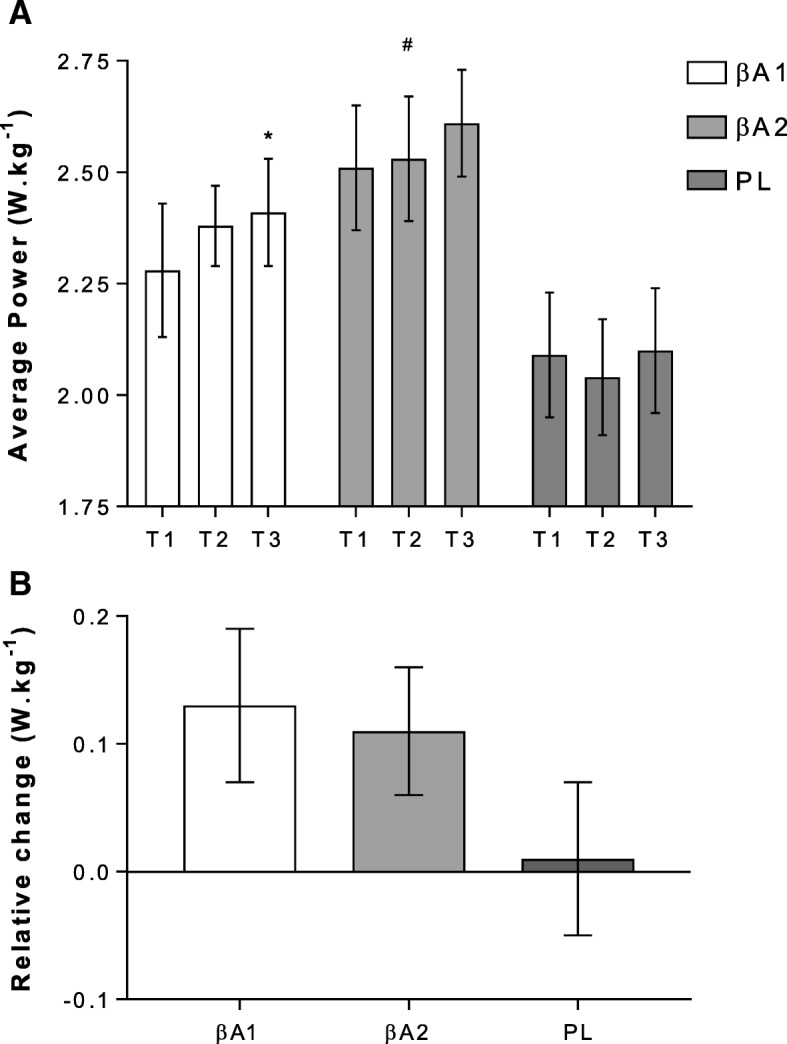
demonstrates effect of beta-alanine (βA) interventions on a) absolute and b) relative power to weight during the 30-min rowing time trial. Abbreviations: βA1 – daily intervention; βA2 – alternate day; PL – placebo. * represents significant difference, from T1, within group only. # represents overall group difference to PL (*P* = 0.04)

Similarly, mean power significantly increased for time only (F = 3.11, *P* = 0.05, ηp^2^ = 0.12) with βA1 from 194.8 ± 18.3 W (T1) to 204.2 ± 15.5 (T3) (*P* = 0.04). No differences were reported within group for βA2 (200.3 ± 9.8 W (T1) to 208.8 ± 8.0 W (T3); *P* > 0.05) or PL (173.0 ± 13.8 W (T1) to 174.8 ± 13.7 W (T3); *P* > 0.05). When adjusted for body-mass, average power output expressed as a) absolute and b) relative change is shown in Fig. 2. A significant time effect was observed for changes in average power to weight (W·kg^− 1^) between T1 and T3 (F = 3.35, *P* = 0.04, ηp^2^ = 0.12) for βA1 only (2.28 ± 0.15 W·kg^− 1^ to 2.41 ± 0.12; *P* = 0.03). A significant between group main effect was reported (F = 3.53, *P* = 0.04, ηp^2^ = 0.12), with post-hoc analysis indicating an overall difference between βA2 and PL only (*P* = 0.04). However, no group x time interactions were shown for absolute changes in average power to weight ratio (F = 1.12, *P* = 0.36, ηp^2^ = 0.09). When data was expressed as relative change in average power, no significant between group differences were observed (F = 1.31, *P* = 0.29); despite small improvements of 0.13 ± 0.06 W·kg^− 1^ for βA1 and 0.11 ± 0.05 W·kg^− 1^ for βA2, in contrast to negligible changes of 0.01 ± 0.06 W·kg^− 1^ for PL.

#### 30-min time trial- average physiological variables and perceived effort

Average HR significantly increased by T3 (F = 4.22, *P* = 0.02, ηp^2^ = 0.15) for βA1 only (175 ± 3 to 180 ± 2 b·min^− 1^; *P* = 0.01), however RPE was maintained throughout the intervention (average RPE: 7.7 ± 0.2 (T1), 7.8 ± 0.2 (T2), 7.8 ± 0.3 (T3) *P* > 0.05). In contrast, average RPE significantly increased by T3 (F = 5.12, *P* = 0.01, ηp^2^ = 0.18) for both βA2 (7.1 ± 0.5 (T1) to 7.7 ± 0.4 (T3); *P* = 0.04) and PL (6.5 ± 0.4 (T1) to 7.3 ± 0.03 (T3); *P* = 0.01). Average VO_2_ was maintained across all trials with no group x time interactions reported (βA1: 3.12 ± 0.12 L·min^− 1^ (T1) to 3.14 ± 0.10 (T3); βA2: 3.17 ± 0.10 L·min^− 1^ (T1) to 3.24 ± 0.14 (T3); PL: 2.93 ± 0.13 L·min^− 1^ (T1) to 2.86 ± 0.14 (T3); *P* > 0.05).

#### 30-min time trial- Split data (0–10 min)

No group x time interactions were shown for overall 0-10 min time trial performance (F = 1.17, *P* = 0.34, ηp^2^ = 0.089; Table 3). A significant effect was shown for time only (F = 8.27, *P* = 0.001, ηp^2^ = 0.256), with βA1 significantly increasing between T1-T2 (*P* = 0.03), T1-T3 (*P* = 0.004) representing a 3.65% (T1-T2) and 4.52% (T1-T3) distance increase, respectively. Likewise, βA2 also increased distance covered significantly by 2.55% from T2-T3 (*P* = 0.01). Accompanying these effects for distance, time effects (F = 4.77, *P* = 0.01, ηp^2^ = 0.166) for absolute power was observed in βA1 only between T1-T2 (*P* = 0.03) and T1-T3 (*P* = 0.01). These represented a 14.04 and 14.61% increase in watts, respectively. Time effects (F = 5.37, *P* = 0.01, ηp^2^ = 0.166) for power to weight was observed in βA1 only, between T1-T2 (*P* = 0.02) and T1-T3 (*P* = 0.01). These represented a 6.20 and 6.75% increase in W·kg^− 1^, respectively.

**Table 3:** Average performance, cardio-respiratory and perceived exertion data for initial 10-min split (0–10 min) during the 30-min TT

	βA1			βA2			PL		
Variable	T1	T2	T3	T1	T2	T3	T1	T2	T3
Distance (m)	2426 ± 87	2503 ± 56^**#**^	2525 ± 61^*^	2483 ± 45	2481 ± 40	2544 ± 42^b^	2318 ± 89	2336 ± 71	2363 ± 74
Power (W)	185.3 ± 21.1	203.1 ± 15.5^#^	204.4 ± 16.4^*^	197.2 ± 10.6	197.9 ± 9.3	209.2 ± 9.2	159.8 ± 20.1	161.7 ± 15.5	165.7 ± 17.0
Power (W·kg^− 1^)	2.16 ± 0.19	2.38 ± 0.12^#^	2.41 ± 0.14^*^	2.47 ± 0.15	2.48 ± 0.14	2.62 ± 0.13	1.93 ± 0.22	1.94 ± 0.17	1.99 ± 0.19
HR (b·min^− 1^)	164 ± 4	171 ± 4	172 ± 3^*^	159 ± 5	165 ± 5	166 ± 4^*^	159 ± 7	161 ± 6	159 ± 6
RPE	6.1 ± 0.4	6.3 ± 0.2	6.1 ± 0.5	5.1 ± 0.6	5.1 ± 0.4	6.2 ± 0.5	4.4 ± 0.5	4.9 ± 0.6	5.0 ± 0.5
V_E_ (L·min^− 1^)	78.6 ± 6.4	81.6 ± 2.8	84.0 ± 4.0	76.4 ± 2.7	77.2 ± 2.6	77.8 ± 2.2	71.8 ± 4.4	73.8 ± 4.4	72.5 ± 3.7
VO_2_ (L·min^− 1^)	3.03 ± 0.26	3.07 ± 0.16	3.23 ± 0.23	2.90 ± 0.15	3.16 ± 0.16	3.13 ± 0.17	2.65 ± 0.18	2.50 ± 0.21	2.47 ± 0.24
VCO_2_ (L·min^− 1^)	3.05 ± 0.26	3.04 ± 0.16	3.12 ± 0.20	3.13 ± 0.20	2.95 ± 0.15	3.18 ± 0.21	2.89 ± 0.27	2.76 ± 0.22	2.74 ± 0.19
RER	0.96 ± 0.02	0.91 ± 0.04	0.89 ± 0.05	0.93 ± 0.05	0.90 ± 0.04	0.82 ± 0.04	0.89 ± 0.03	0.91 ± 0.03	0.95 ± 0.02

Data in Table 3 refers to the first 10-min split of the 30-min TT. Time points denoted by T1-T3. Abbreviations: *HR* heart rate, *RPE* rating of perceived exertion, *V*_*E*_ minute ventilation, *VO*_*2*_ absolute oxygen uptake, *VCO*_*2*_ absolute carbon dioxide, *RER* respiratory exchange ratio. # = significantly different T1-T2 within group only (*P* < 0.03); * = significant difference within group T1-T3 (*P* < 0.05); b = significant difference T2-T3 within group only (*P* = 0.01)

A significant effect was observed for changes in HR (b·min^− 1^) between T1 and T3 (F = 4.992, *P* = 0.01, ηp^2^ = 0.17) for βA1 (164 ± 4 to 172 ± 3 b·min^− 1^; *P* = 0.02) and βA2 (159 ± 5 b·min^− 1^ to 166 ± 4; *P* = 0.05), but not PL (*P* > 0.05). In contrast, average RPE (F = 1.607, *P* = 0.19, ηp^2^ = 0.118), V_E_ (F = 0.410, *P* = 0.41, ηp^2^ = 0.036), VO_2_ (F = 1.398, *P* = 0.25, ηp^2^ = 0.104), VCO_2_ (F = 0.384, *P* = 0.82, ηp^2^ = 0.031) and RER (F = 2.234, *P* = 0.08, ηp^2^ = 0.157) were maintained across all trials with no significant interactions reported.

#### 30-min time trial- Split data (11–20 min)

No group x time interactions were shown during the second 10-min split (F = 0.694, *P* = 0.60, ηp^2^ = 0.055; Table 4). Within group however a significant time effect was demonstrated (F = 3.987, *P* = 0.03, ηp^2^ = 0.257), with βA1 increasing distance rowed by 54 ± 14 m between T1-T3 (*P* = 0.01). Other performance variables such as absolute power (F = 1.283, *P* = 0.29, ηp^2^ = 0.051) and power to weight (F = 1.177, *P* = 0.32, ηp^2^ = 0.073) failed to reach significance overall. Average HR (F = 1.893, *P* = 0.16, ηp^2^ = 0.073), V_E_ (F = 0.959, *P* = 0.39, ηp^2^ = 0.038), VO_2_ (F = 0.780, *P* = 0.46, ηp^2^ = 0.031), VCO_2_ (F = 1.115, *P* = 0.34, ηp^2^ = 0.044), RPE (F = 2.971, *P* = 0.07, ηp^2^ = 0.205), and RER (F = 0.079, *P* = 0.93, ηp^2^ = 0.003) were maintained across all trials with no significant interactions reported.

**Table 4:** Average performance, cardio-respiratory and perceived exertion data for mid 10-min split (11–20 min) during the 30 min TT

	βA1			βA2			PL		
Variable	T1	T2	T3	T1	T2	T3	T1	T2	T3
Distance (m)	2449 ± 67	2478 ± 56	2502 ± 60*	2458 ± 35	2477 ± 36	2497 ± 34	2365 ± 63	2373 ± 47	2362 ± 60
Power (W)	189.8 ± 16.2	199.9 ± 14.3	201.4 ± 16.7	197.6 ± 10.2	199.4 ± 9.1	201.1 ± 7.9	168.8 ± 14.3	168.7 ± 11.9	169.4 ± 14.7
Power (W·kg^− 1^)	2.23 ± 0.13	2.35 ± 0.10	2.37 ± 0.14	2.47 ± 0.15	2.50 ± 0.14	2.52 ± 0.13	2.04 ± 0.14	2.04 ± 0.14	2.03 ± 0.14
HR (b·min^− 1^)	174 ± 4	177 ± 3	178 ± 2	169 ± 4	173 ± 53	172 ± 4	171 ± 5	170 ± 4	170 ± 4
RPE	7.7 ± 0.3	7.7 ± 0.2	7.6 ± 0.3	6.9 ± 0.7	7.0 ± 0.6	7.7 ± 0.4	6.2 ± 0.5	6.9 ± 0.4	7.4 ± 0.4
V_E_ (L·min^− 1^)	88.8 ± 6.7	91.0 ± 4.0	88.6 ± 5.3	83.5 ± 2.5	78.4 ± 3.9	82.5 ± 2.8	83.8 ± 2.2	78.5 ± 3.4	80.1 ± 3.1
VO_2_ (L·min^− 1^)	3.28 ± 0.26	3.38 ± 0.23	3.60 ± 0.44	3.17 ± 0.13	3.14 ± 0.14	3.10 ± 0.16	3.07 ± 0.13	2.69 ± 0.10	3.00 ± 0.19
VCO_2_ (L·min^− 1^)	3.20 ± 0.22	3.30 ± 0.12	3.26 ± 0.20	3.30 ± 0.16	2.97 ± 0.14	3.20 ± 0.19	3.29 ± 0.25	2.98 ± 0.25	3.11 ± 0.25
RER	1.04 ± 0.02	1.02 ± 0.01	0.99 ± 0.03	0.97 ± 0.04	0.97 ± 0.02	0.97 ± 0.04	1.00 ± 0.02	1.03 ± 0.01	1.04 ± 0.03

Data in Table 4 refers to the second 10-min split of the 30-min TT. Time points denoted by T1-T3. *HR* heart rate, *RPE* rating of perceived exertion, *V*_*E*_ minute ventilation, *VO*_*2*_ absolute oxygen uptake, *VCO*_*2*_ absolute carbon dioxide, *RER* respiratory exchange ratio. * = significant difference within group T1-T3 (*P* = 0.01)

#### 30-min time trial- Split data (21–30 min)

No group x time interactions were shown for overall 21–30 min time trial distance covered (F = 2.027, *P* = 0.21, ηp^2^ = 0.144; Table 5). Likewise, no effects were shown for time (F = 1.594, *P* = 0.12, ηp^2^ = 0.062) in distance covered or other performance variables (*P* > 0.05). Average HR (F = 0.841, *P* = 0.41, ηp^2^ = 0.034), V_E_ (F = 0.959, *P* = 0.39, ηp^2^ = 0.038), VO_2_ (F = 1.649, *P* = 0.20, ηp^2^ = 0.064), VCO_2_ (F = 1.850, *P* = 0.18, ηp^2^ = 0.070) RPE (F = 2.882, *P* = 0.66, ηp^2^ = 0.107) and RER (F = 0.803, *P* = 0.0.76, ηp^2^ = 0.032) were maintained across trials with no significant group x time interactions reported.

**Table 5:** Average performance, cardio-respiratory and perceived exertion data for final 10-min split (21–30 min) during the 30-min TT, including endpoint [La-]_b_

	βA1			βA2			PL		
Variable	T1	T2	T3	T1	T2	T3	T1	T2	T3
Distance (m)	2533 ± 49	2543 ± 43	2552 ± 56	2526 ± 34	2537 ± 39	2534 ± 39	2503 ± 35	2431 ± 44	2503 ± 35
Power (W)	213.4 ± 16.6	206.0 ± 12.1	206.7 ± 14.3	206.2 ± 9.4	207.0 ± 9.5	217.9 ± 10.9	193.2 ± 9.2	180.2 ± 10.9	190.8 ± 11.0
Power (W·kg^−1^)	2.51 ± 0.12	2.43 ± 0.08	2.44 ± 0.11	2.58 ± 0.13	2.60 ± 0.14	2.72 ± 0.14	2.34 ± 0.09	2.17 ± 0.11	2.30 ± 0.10
HR (b.min^− 1^)	185 ± 5	189 ± 3	190 ± 3	183 ± 3	183 ± 4	182 ± 3	185 ± 3	184 ± 2	185 ± 3
RPE	9.3 ± 0.2	9.4 ± 0.2	9.7 ± 0.2	9.2 ± 0.3	9.2 ± 0.3	9.3 ± 0.3	8.8 ± 0.4	9.2 ± 0.3	9.6 ± 0.2
V_E_ (L·min^−1^)	100.4 ± 3.5	105.0 ± 3.3	103.2 ± 3.2	95.0 ± 1.8	96.6 ± 3.5	99.7 ± 2.2	100.6 ± 2.2	98.8 ± 3.0	100.1 ± 2.7
VO_2_ (L·min^− 1^)	3.68 ± 0.26	3.75 ± 0.30	3.63 ± 0.25	3.45 ± 0.12	3.12 ± 0.27	3.50 ± 0.16	3.29 ± 0.18	3.02 ± 0.14	3.33 ± 0.28
VCO_2_ (L·min^−1^)	3.75 ± 0.14	3.82 ± 0.16	3.77 ± 0.15	3.70 ± 0.16	3.35 ± 0.22	3.77 ± 0.19	3.91 ± 0.27	3.58 ± 0.22	3.94 ± 0.16
RER	1.10 ± 0.01	1.11 ± 0.03	1.13 ± 0.01	1.09 ± 0.02	1.07 ± 0.02	1.10 ± 0.01	1.11 ± 0.01	1.10 ± 0.02	1.12 ± 0.02
[La-]_b_ (mmol·L^− 1^)	10.7 ± 0.8	9.3 ± 0.6	10.0 ± 0.8	9.0 ± 1.0	8.4 ± 0.9	8.8 ± 1.1	9.4 ± 0.8	9.8 ± 0.9	9.1 ± 0.1

Data in Table 5 refers to the final 10-min split of the 30-min TT. Time points denoted by T1-T3. *HR* heart rate, *RPE* rating of perceived exertion, *V*_*E*_ minute ventilation, *VO*_*2*_ absolute oxygen uptake, *VCO*_*2*_ absolute carbon dioxide, *RER* respiratory exchange ratio, *[La-]*_*b*_ post test blood lactate. No differences reported

#### Overall sprint performance

No significant between-group effects existed at T1 with all groups rowing 166.0 ± 2.5 m (F = 2.325, *P* = 0.07, ηp^2^ = 0.162; Table 6). Following treatment with their respective intervention, no significant group x time (F = 2.325, *P* = 0.07, ηp^2^ = 0.162), or time (F = 1.936, *P* = 0.16, ηp^2^ = 0.075) effects were observed at any time-point for distance covered. Likewise, power (F = 1.961, *P* = 0.15, ηp^2^ = 0.076), power to weight (F = 1.251, *P* = 0.30, ηp^2^ = 0.050), HR (F = 1.241,* P* = 0.30, ηp^2^ = 0.049), RPE (F = 3.920, *P* = 0.26, ηp^2^ = 0.140) and [La-]_b_ (F = 0.759, *P* = 0.46, ηp^2^ = 0.032) failed to reach significance.

**Table 6:** Average sprint performance data across T1-T3

	βA1			βA2			PL		
Variable	T1	T2	T3	T1	T2	T3	T1	T2	T3
Distance (m)	165 ± 4	165 ± 4	164 ± 4	159 ± 3	160 ± 3	161 ± 4	163 ± 3	159 ± 2	163 ± 4
Power (W)	355.4 ± 13.8	355.4 ± 13.8	352.7 ± 14.5	333.5 ± 10.9	339.1 ± 12.1	342.2 ± 13.5	351.0 ± 13.1	335.0 ± 9.5	348.2 ± 13.9
Power (W·kg^−1^)	4.21 ± 0.06	4.21 ± 0.12	4.20 ± 0.13	4.16 ± 0.12	4.25 ± 0.15	4.27 ± 0.16	4.20 ± 0.08	4.06 ± 0.13	4.20 ± 0.11
HR (b·min^− 1^)	170 ± 5	177 ± 4	178 ± 3	170 ± 3	172 ± 5	174 ± 3	170 ± 5	168 ± 4	170 ± 4
RPE	9.0 ± 0.1	9.3 ± 0.1	9.3 ± 0.2	8.8 ± 0.2	9.0 ± 0.2	9.3 ± 0.2	8.7 ± 0.4	8.7 ± 0.4	9.1 ± 0.2
[La-]_b_	10.7 ± 1.0	9.6 ± 0.6	10.0 ± 0.9	9.1 ± 0.9	8.8 ± 1.0	9.2 ± 1.1	8.9 ± 0.9	8.9 ± 1.0	8.7 ± 0.9

Data refers to mean values across the 3 repeated sprints at time-points T1-T3 and experimental condition. No significant differences reported (*P* > 0.05)

## Discussion

The aim of the current randomised controlled trial was to observe the effects of two separate, 28-day, βA dosing strategies (matched for total overall dose), on 30-min rowing time trial and subsequent anaerobic sprint performance. A recent 2018 ISSN Position Stand [40] stated there is ‘strong evidence’ to support the efficacy of βA as an ergogenic aid, with research demonstrating positive influences over short duration performance [11–13] and capacity [3] measures alike. However, the primary finding of this study indicates that βA does not appear to offer significant benefits to sustained endurance performance, as assessed via a 30-min time trial when compared to placebo. These findings concur with an earlier 2015 ISSN Position Stand [14] indicating that βA consumption “does not demonstrate a consistent positive effect” on events lasting beyond a 25-min timeframe. It is, however, noteworthy that within-group improvements were observed for mean distance covered, average power and average power to weight ratio when participants consumed βA daily, increasing by 2.6, 14.6 and 14.9%, respectively over the intervention period. This may likely be the result of improved effort (distance covered and power output (including relative to mass)) in the first 10-min split. In the second 10-min split, whilst a within group increase was observed for distance covered (T1-T3) for βA1, power output did not significantly change, potentially indicating a diminishing effect with time trial duration.

The findings from the current study support previous research on 10-km running performance [41], whereby a mean reduction in time taken to complete 10-km (Pre = 3441 ± 327, Post = 3209 ± 271 s) was observed within the βA group only. However, it should be noted that the participants mean baseline 10-km time was a conservative 57.35 min, suggesting that results may have been confounded by the experience of the runners. Nonetheless, beyond this study there exists a lack of results during comparable (≥30- min) protocols. More specifically, the current split data support Saunders and colleague’s meta-analysis in suggesting that the strength of evidence supporting βA’s efficacy does not extend to longer duration (> 10-min) events [9] (despite the fact that many athletes use βA for endurance-based events). This same review noted that findings were potentially confounded by an absence of research investigating longer duration events [14] and the predominant use of incremental tests [9], two issues the current study attempted to circumvent. Therefore, whilst these data could be interpreted as providing preliminary support for βA’s potential to facilitate small scale improvements, when compared to a placebo there appears to be no significant benefit of consuming βA in the short term for longer duration aerobic exercise.

Regarding the anaerobic sprint data, no significant interaction effects were observed for any variables. This was unexpected as previously Suzuki et al. [42] noted a strong positive correlation between muscle carnosine concentration and Wingate performance. Likewise, rowers have exhibited greater muscle carnosine concentration and buffering capacity when compared with both marathoners and non-trained controls [43] . Therefore, it had been hypothesised that facilitating elevated muscle carnosine via βA consumption could have a significant effect on rowing sprint performance. One explanation for the lack of effect in the current study may have been the different exercise modality (rower vs cycle) employed. When cycling, the smaller muscle mass engaged may be more susceptible to localised muscular acidosis [44], providing a more optimal environment for βA’s effects to augment. This theory could help support previous research whereby mean power was significantly increased during a 30-s sprint following an endurance cycling event [10], proposing that βA may only facilitate ergogenic effects when there is an increased requirement to protect the ‘*milieu interieur*’ from homeostatic perturbations caused by supra-maximal levels of intracellular acidosis. However, whilst the current cohorts [La^−^]_b_ were clearly elevated following each sprint bout, the mean [La^−^]_b_ post time trial and during associated sprint efforts for βA1 were not significantly affected by βA consumption compared to PL, possibly suggesting an absence of meaningful carnosine facilitated buffering at the current dosage.

A novel aspect of this study was the inclusion of an alternate day dosing strategy (at a matched total dose). Previous research has suggested that the primary facilitator of muscle carnosine concentration is the total dose consumed, not factors such as baseline content or daily dose [29]. The current data does not support this hypothesis given the lack of significant findings in overall power, power to weight and distance covered at all time-points for βA2. Likewise, despite an isolated increase from T2-T3 in distance rowed during the first 10-min of the time trial, all other split data recorded support a lack of effectiveness when βA is not consumed daily. It is noteworthy, however, that although body composition (including fat-free mass) was not significantly different between groups, two individuals within βA2 reported body-fat percentages below 10% in contrast to other participants. Furthermore, fat-free mass ranged from 63.3–77.0 kg within βA2 (in contrast to 62.6–85.3 kg for βA1 and 60.3–85.0 kg for PL). Although unlikely, based on individual performance differences and average power (W and W·kg^− 1^), it is feasible that one explanation for a lack of significant findings with βA2 may have been influenced as a result of variances in lean muscle mass.

Other reasons for variation caused by dosing may reside in the pharmacokinetics of βA, with previous research [45] exhibiting a large variation between participants when given an equimolar amount (1400 mg) of the supplement. Alternatively, the consistency of daily consumption could potentially produce a more favourable environment for the bio-availability and subsequent augmentation of carnosine. Future research is required to investigate both the pharmacokinetics of βA beyond a single bolus and the potential mechanistic pathways that facilitate more optimal carnosine augmentation.

Limitations of this study include a lack of baseline and/or temporal measures of skeletal muscle carnosine concentrations. However, the dosing strategy utilised was equal to or greater than preceding work that has quantified muscle carnosine content [2, 46]. Furthermore, whilst [La^−^]_b_, RPE and HR were recorded throughout the test, blood pH was not. Subsequently, it must be conceded that the capacity to assess or infer whether effects were associated with carnosine directed (pH) buffering are limited. Additionally, due to a commonly reported side effect of βA (paraesthesia [1]), participants within either experimental arm of this trial may have become aware they were consuming the supplement. However, instructions to consume small (800 mg) individual doses, with food and regular verbal questioning at each visit indicated this was highly unlikely, with no cases of paraesthesia being reported. Future research should adopt dosing protocols that deliver βA in a sustained-release (SR) formula which may negate this issue [47, 48].

Beyond the delivery method of βA, previous research demonstrated that at an average daily dose of 5.2 g·day^− 1^ for four-weeks is not sufficient to maximise muscle carnosine content [3]. More recently this claim has been supported by research that supplied participants with 6.4 g·day^− 1^ of βA each day for 24 weeks, observing gene expression, muscle carnosine content and cycling capacity (CCT110%) [49]. Interestingly, this study’s primary observations was that muscle carnosine increased in the experimental group at all time-points with no change in PL, thus, investigations into the effects of higher dose (> 4 g·day^− 1^ [14]) or duration (24+ weeks) interventions with βA during endurance events are merited. Finally, whilst βA may provide some ergogenic influence in young, well-trained athletes, its effects within individuals who have reduced carnosine content (untrained, vegan/vegetarian or master’s athletes) [31] may be worthwhile, due to potentially amplified effects.

## Conclusion

In conclusion, regardless of dose strategy, when compared to placebo, βA does not enhance sustained aerobic performance or subsequent high intensity efforts. However, the within-group finding that daily βA use increased 30-min rowing time trial performance warrants further investigation. The inclusion of higher dosing strategies (> 2.4 g·day^− 1^) for longer periods (> 28 days) should also be considered.

## Abbreviations

[La^−^]_b_: Blood lactate
P:W: power to weight ratio
SR: Sustained release
βA: Beta-alanine
βA1: Beta-alanine daily intervention strategy
βA2: Beta-alanine alternate day strategy
